# Givinostat reduces adverse cardiac remodeling through regulating fibroblasts activation

**DOI:** 10.1038/s41419-017-0174-5

**Published:** 2018-01-25

**Authors:** Marika Milan, Valentina Pace, Fabio Maiullari, Maila Chirivì, Denisa Baci, Silvia Maiullari, Luca Madaro, Sonia Maccari, Tonino Stati, Giuseppe Marano, Giacomo Frati, Pier Lorenzo Puri, Elena De Falco, Claudia Bearzi, Roberto Rizzi

**Affiliations:** 1 Institute of Cell Biology and Neurobiology (IBCN), National Research Council of Italy (CNR), Monterotondo Scalo, Rome, 00015 Italy; 2 Operational Research Unit, Fondazione di Ricerca e Cura Giovanni Paolo II, Largo Gemelli 1, Campobasso, Italy; 3 IRCCS Fondazione Santa Lucia, Rome, 00142 Italy; 4 Centro di Riferimento per la Medicina di Genere Istituto Superiore di Sanità Viale Regina Elena, 299 Roma, Italy; 5 Department of Medical Surgical Sciences and Biotechnologies, Sapienza University of Rome, 04100 Latina, Italy; 6 Department of AngioCardioNeurology, IRCCS NeuroMed, 86077 Pozzilli (IS), Italy; 7 Development, Aging and Regeneration Program, Sanford Burnham Prebys Medical Discovery Institute, La Jolla, CA 92037 USA

## Abstract

Cardiovascular diseases (CVDs) are a major burden on the healthcare system: indeed, over two million new cases are diagnosed every year worldwide. Unfortunately, important drawbacks for the treatment of these patients derive from our current inability to stop the structural alterations that lead to heart failure, the common endpoint of many CVDs. In this scenario, a better understanding of the role of epigenetics – hereditable changes of chromatin that do not alter the DNA sequence itself – is warranted. To date, hyperacetylation of histones has been reported in hypertension and myocardial infarction, but the use of inhibitors for treating CVDs remains limited. Here, we studied the effect of the histone deacetylase inhibitor Givinostat on a mouse model of acute myocardial infarction. We found that it contributes to decrease endothelial-to-mesenchymal transition and inflammation, reducing cardiac fibrosis and improving heart performance and protecting the blood vessels from apoptosis through the modulatory effect of cardiac fibroblasts on endothelial cells. Therefore, Givinostat may have potential for the treatment of CVDs.

## Introduction

Cardiac remodeling and fibrosis are compensatory mechanisms consequent to ischemic events^1^ and they strictly determine the clinical outcome. Indeed, after an ischemic event there is an initial phase of remodeling and recovery, during which damaged cardiomyocytes (CMs) are replaced by new cells; however, this leads to a secondary phase characterized by fibrosis^2^, a process that, when unchecked, causes the generation of excessive remodeling of the cardiac extracellular matrix, oxidative stress, and inflammation within the ischemic microenvironment^3^. Although fibrosis and inflammation are initially beneficial^4^, they become detrimental in the long term, suggesting that therapy should aim for the control rather than the suppression of both events.

Among the biological and molecular mechanisms involved in the adaptive response to a cardiac insult, histone deacetylase (HDAC)-mediated epigenetics processes are receiving a special attention. HDACs are common enzymes regulating deacetylation of core histones and are strictly correlated to the regulation of homeostatic gene expression of vascular and cardiac cell populations, including stem cell commitment^5^. More importantly, abnormal acetylation of core histones, a process likely linked to environmental factors, has been associated with major cardiovascular diseases^6^. After a cardiac insult, HDACs activity is enhanced, resulting in increased proliferation, migration, and apoptosis of adventitial fibroblasts (FBs), endothelial cells (ECs), and muscle cells, as well as stimulation of macrophage (MP) activation and phenotype switching^7^ suggesting an involvement of HDACs in driving the response to injury and remodeling even through the early inflammatory phase. A wide range of molecules have been tested in their ability to inhibit HDACs^8^. Pan- and selective HDAC inhibitors (HDACi) have been shown to preserve cardiac function in disease states by exerting an anti-inflammatory effect and reducing cardiac hypertrophy and fibrosis^9,10^ through signals mainly targeting oxidases and/or specific kinases^11,12^. Despite this, epigenetics-based therapies are still limited in the cardiovascular field and the use of the HDACi has still to be clearly elucidated, including safety and long-term effects.

Givinostat (ITF2357) is a powerful pan-HDACi that has gained considerable attention due to its varied applicability, efficacy, and safety in humans. Described in 2005^13^, Givinostat is currently being tested in clinical trials on different diseases^14–18^. The drug has been shown to decrease tnf-α, il-6, and il-1 levels, producing a striking reduction of the inflammatory response in combination with pro-angiogenic effects. To date, the effects of Givinostat on cardiac diseases remain to be verified, but studies on Duchenne muscular dystrophy (DMD) suggest that the HDACi might act beneficially on the cardiac muscle as well^18^.

Therefore, we decided to study the biological and functional efficacy of Givinostat on acute myocardial infarction (AMI). We found that the drug improved post-AMI heart function by hindering the development of fibrosis, likely via a mechanism targeting endothelial-to-mesenchymal transition (EndMT). Thus, Givinostat holds promise for the treatment of cardiovascular diseases.

## Results

To test the efficacy of Givinostat on heart failure, 10-week-old C57 mice underwent surgery to induce AMI by permanent ligation of the left descending coronary artery: one group of mice was treated daily with Givinostat for 1, 3, 7, 15, or 30 days, while a control group was administered with saline. At the end of the treatments, mice were killed.

**Fig. 1:**
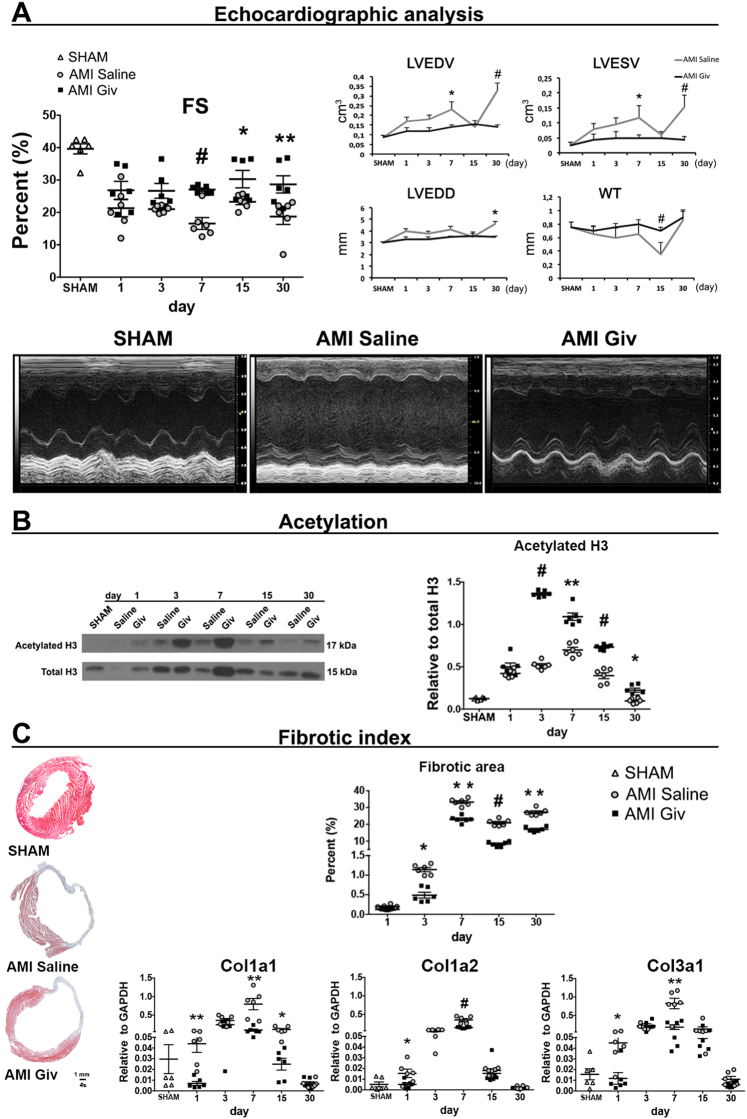
Givinostat effect on infarcted heart and cardiac fibrosis. **a** Echocardiographic measurements indicate an amelioration in fractional shortening (FS), left ventricular end diastolic volume (LVEDV), left ventricular end systolic volume (LVESV), left ventricular end diastolic diameter (LVEDD) and wall thickness (WT). Representative M-mode images of control mice (left panel) and after injection with saline (middle panel) or Givinostat (right panel) in AMI mice. **b** Western blot analysis shows an increased acetylated H3 protein levels in the whole heart tissue after Givinostat administration compared to saline group. **c** After Givinostat treatment, four sections per mouse were analyzed by Masson’s trichrome staining. Scale bar represent 1 mm. Percentage of fibrotic area: fibrotic area/total area × 100. Quantitative RT-PCR illustrates the Givinostat effect on the expression of fibrosis-related genes (Col1a1, Col1a2 and Col3a1) at different time points. *N* = 6 mice for time point. Error bars represent ± SEM. Student’s *t* test, *p < 0.05, **p < 0.01, ^#^*p* < 0.001

Cardiac performance was evaluated by echocardiography. Saline administered mice suffered progressive declines in fractional shortening (FS) as expected (Fig. 1a). Interestingly, Givinostat treatment significantly improved the percentage of FS at day 7, 15, and 30 (Fig. 1a) compared to controls. Left Ventricular End Diastolic Volume (LVEDV), Left Ventricular End Systolic Volume (LVESV), Left Ventricular End Diastolic Diameter (LVEDD), and wall thickness (WT) measurements confirmed modulation of cardiac remodeling. There were no differences in the WT parameter, which was calculated as Frontal Wall thickness+Posterior Wall thickness/2, between the two groups at day 30. Indeed, the hypertrophy of the back wall of control animals is counteracted by the reduced loss of muscle tissue in treated group (Fig. 1a; Table 1).

**Table 1:** Echocardiographic parameters

day		1		3		7		15		30	
Parameters	SHAM	Saline	Giv	Saline	Giv	Saline	Giv	Saline	Giv	Saline	Giv
FS %	38.6 ± 1.5	21.3 ± 2.6	26.8 ± 2.7	21 ± 0.4	26.6 ± 2.2	16.5 ± 1.8	27 ± 0.4*	23.2 ± 0.8	30.2 ± 2.7*	18.7 ± 2.4	28.6 ± 2.6**
EF %	75.6 ± 2	49.8 ± 6.1	60 ± 4	51 ± 0.8	58.8 ± 3.4	39.1 ± 1.4	62.6 ± 0.7*	57.2 ± 0.8	62.6 ± 3.3	48.6 ± 6.3	63.8 ± 3.8*
IVSd (mm)	0.6 ± 0.08	0.6 ± 0.1	0.6 ± 0.1	0.5 ± 0.01	0.6 ± 0.06	0.5 ± 0.05	0.7 ± 0.08	0.6 ± 0.08	0.5 ± 0.09	0.7 ± 0.1	0.7 ± 0.3
IVSs (mm)	0.9 ± 0.09	0.7 ± 0.07	0.8 ± 0.1	0.7 ± 0.07	0.9 ± 0.09	0.8 ± 0.1	0.9 ± 0.03	0.1 ± 0.2	0.9 ± 0.1	1 ± 0.2	1.1 ± 0.3
LVIDd (mm)	3 ± 0.1	4 ± 0.1	3.3 ± 0.1	3.8 ± 0.2	3.3 ± 0.2	4.1 ± 0.3	3.5 ± 0.1*	3.5 ± 0.2	3.6 ± 0.1	4.6 ± 0.4	3.5 ± 0.1*
LVIDs (mm)	2 ± 0.1	2.9 ± 0.2	2.4 ± 0.2	3.1 ± 0.2	2.5 ± 0.2	3.3 ± 0.4	2.5 ± 0.1*	2.7 ± 0.1	2.5 ± 0.2	3.8 ± 0.4	2.4 ± 0.1*
LVPWd (mm)	0.7 ± 0.09	0.6 ± 0.08	0.5 ± 0.05	0.5 ± 0.06	0.6 ± 0.06	0.5 ± 0.07	0.7 ± 0.1	0.6 ± 0.09	0.6 ± 0.06	0.4 ± 0.09	0.5 ± 0.08
LVPWs (mm)	1 ± 0.1	0.9 ± 0.09	0.7 ± 0.08	0.6 ± 0.06	0.8 ± 0.1	0.7 ± 0.1	1 ± 0.1	0.8 ± 0.1	0.7 ± 0.07	0.5 ± 0.08	0.4 ± 0.09

*FS* fractional shortening, *EF* ejection fraction, *IVSs* intraventricular septum in systole, *IVSd* intraventricular septum in diastole; *LVIDd* left ventricular internal diameter in diastole, *LVIDs* left ventricular internal diameter in systole, *LVPWd* left ventricular posterior wall in diastole, *LVPWs* left ventricular posterior wall in systole

Values are means ± SEM

*N* = 6 for time point.

Notably, Givinostat administration modified the epigenetic state of cardiac tissue, by increasing protein level of acetylated Histone 3 at day 3, 7, 15, and 30 (Fig. 1b). The improvement in the percentage of FS, in treated animals, was histologically accompanied by a decreased percentage of cardiac fibrotic area (Masson’s trichrome) over the time respect to controls (Fig. 1c).

Coherently, quantitative RT-PCR (qRT-PCR) analysis showed a diminished expression of fibrosis-associated genes, including collagen 1a1 (Col1a1), collagen 1a2 (Col1a2) and collagen 3a1 (Col3a1) in the hearts of Givinostat treated mice.

**Fig. 2:**
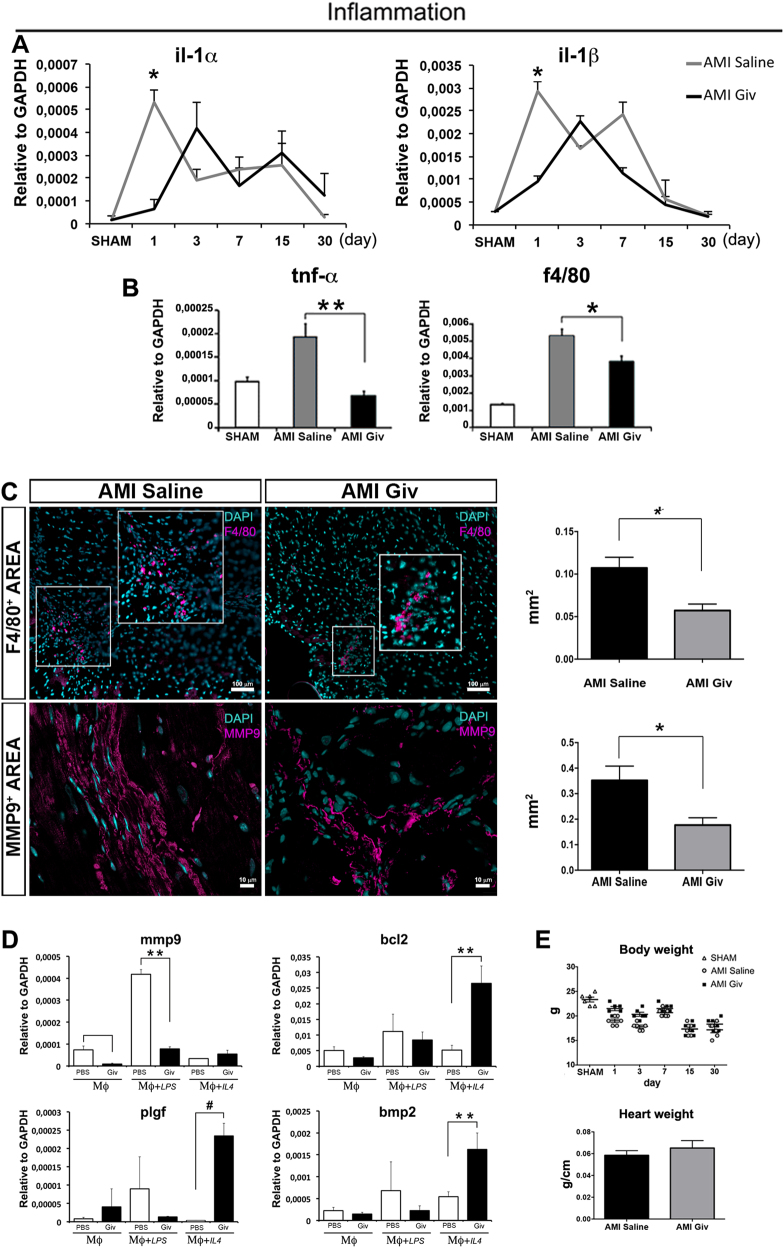
Inflammatory process assays. **a** The graphs display the mRNA levels of interleukins il-1α and il-β after Givinostat administration in infarcted hearts. **b** Expression levels of tnf-α and f4/80 genes in AMI mice treated with Givinostat after 1 day. **c** Sections of AMI mouse heart treated with saline (left panels) and Givinostat (right panels) at day 7 composed of cells positive for F4/80 (magenta, upper images) and MMP9 (magenta, lower images). Scale bars represent 100 and 10 µm respectively. The graphs highlight F4/80 and MMP9 infiltrating areas (mm^2^) normalized to the total area of cardiac tissue, in the animals treated with Givinostat and in the control group. **d** Quantitative RT-PCR demonstrates a reduction of mmp9 expression in M1 macrophages and an overexpression of bcl2, plgf and bmp2 genes in M2 macrophages after Givinostat exposure. **e** Effect of Givinostat on body weight at different time points and on heart weight normalized to the tibia length, at the end of the treatment in AMI mice. *N* = 6 mice for time point. Error bars represent ± SEM. Student’s *t* test, *p < 0.05, **p < 0.01, ^#^p < 0.001

Moreover, inflammatory activity was drastically reduced following treatment with the HDACi. Indeed, the expression of interleukins il-1α and il-1β (Fig. 2a) was significantly decreased one day after Givinostat administration, leading us to speculate that the beneficial effects of the drug may act via modulation of inflammatory cells, such as macrophages (MPs). Expressions of tumor necrosis factor-α (tnf-α) and f4/80 (a macrophage receptor) were significantly reduced in infarcted hearts treated with Givinostat. This outcome was confirmed by histological measurements, where F4/80 and MMP9 positive areas were detected and normalized to the total cardiac section (Fig. 2c).

The above results suggest that the beneficial effect of Givinostat depends, at least partially, on the blunting of the inflammatory process.

We assumed that the cells mediating this HDACi effect were MPs. To test this hypothesis, we isolated the MP population from the bone marrow of mice. One MP group was treated with Lipopolysaccharide (LPS) and another with Interleukin 4 (IL-4) to induce respectively M1 and M2 phenotypes. A third MP group was non-polarized (Naive). All clusters were treated with Givinostat and analyzed after 24 h by qRT-PCR.

The expression of mmp9, a metalloproteinase enhancing migration capacity of MPs in M1 phase through the recall sites, was significantly reduced (Fig. 2d), while, in the M2 phenotype the expression of bcl2, which in MPs guarantees protection from apoptosis (Fig. 2d), was increased after treatment. Givinostat administration in presence of AMI did not alter body and heart weights over the time (Fig. 2e) compare to the control.

In addition, Givinostat treatment significantly increased placenta growth factor (plgf) gene expression, a known angiogenic factor, which in M2 recalls blood vessels to support the regenerative phase (Fig. 2d). Lastly, bone morphogenic protein 2 (bmp-2) increased expression (Fig. 2d) could robustly activate MPs through pSmad1/5/8 signaling pathway generating a positive feedback loop by increasing the expression of angiogenic factors. Data were confirmed by histological analysis (Fig. 2e).

Hence, we isolated primary MPs and CMs from the C57 neonatal mice to perform co-culture experiments to assess the effect of the latter on the former (Supplementary Figure 1). The in vitro experiment involved the gene expression analysis of CMs alone or co-cultured with the MPs-naive or -M1 or -M2 phenotypes after Givinostat administration. MPs-M2 exposed to Givinostat increased the maturation of neonatal CMs, suggesting that MPs can influence CM fate. Moreover, a very interesting effect consisted in the reduction of the Caspase 3 (casp3) expression, which hints a protective role from apoptosis confirmed by bcl2 expression. In contrast, direct exposure of CMs to Givinostat resulted in induction of early cardiac genes implying a re-activation of the cardiac embryonic program, and suggesting the triggering of the hypertrophic pathway, which is prevented if the effect of Givinostat is mediated by the MPs-M2.

**Fig. 3:**
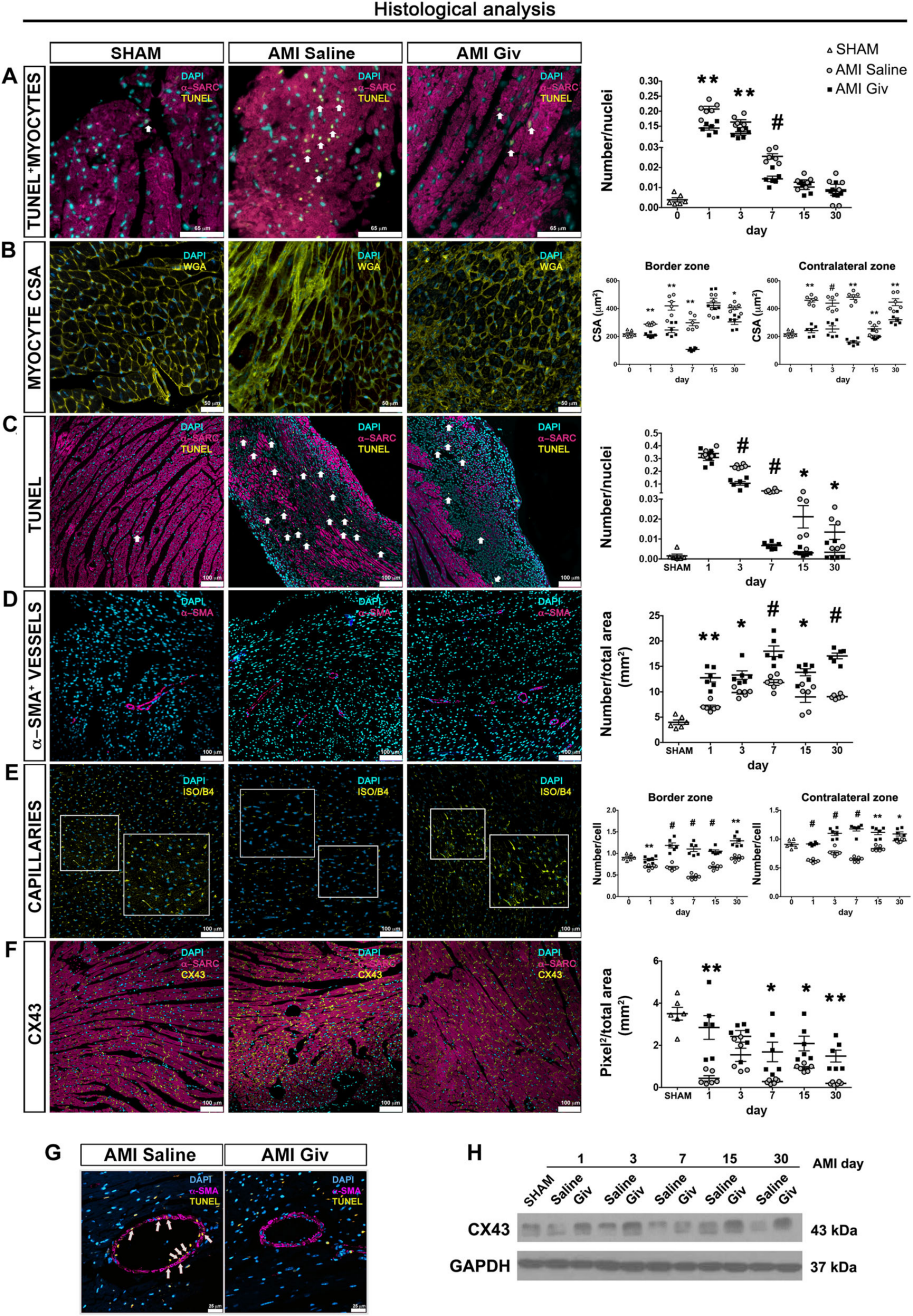
Histological analysis. **a** Apoptotic cardiomyocytes (α-SARC, magenta) were detected by TUNEL labeling (yellow) in SHAM and AMI mice at day 7. Scale bar represent 65 μm. The graph illustrates the ratio between the number of double positive cells (α-SARC^+^ and TUNEL^+^ cells) and the nuclei at different time points. **b** Cross sectional area (CSA) in the border zone at day 30 stained with wheat germ agglutinin (WGA, yellow). Scale bar represent 50 μm. Plot indicates myocytes CSA (μm^2^) in both the border and the contralateral zone at different time points. **c** Apoptotic cells were detected by TUNEL labeling (yellow) at day 7. Scale bar represent 100 μm. The graph indicates the ratio between the number of whole TUNEL positive cells and the nuclei at different time points. **d** α-SMA positive vessels labeled in magenta after 30 days. Scale bar represent 100 μm. The corresponding diagram shows the ratio number of α-SMA positive vessels on total area (mm^2^) at different time points. **e** Isolectin B4 positive capillaries (yellow) in the border zone at day 30. Scale bar represent 100 μm. Graphs explain the proportion number of ISO/B4 positive capillaries/cell number in the border and the contralateral zone at different time points. **f** Staining for Cx43 (yellow) in the border zone of the infarcted area at day 30. Scale bar represent 100 μm. Cx43 quantification, expressed as pixel^2^/total area, is elucidated in the side chart. For Cx43 quantification method details see Materials and methods section and supplementary Figure S5. **g** Heart cross section of vessel composed of smooth muscle cells (α-SMA, magenta) and apoptotic cells (yellow) highlighted by arrows (white). Scale bar represent 25 μm. **h** Western blot analysis for Cx43 in AMI Givinostat mice vs. AMI Saline group at different time points. *N* = 6 mice for time point. Error bars represent ± SEM. Student’s *t* test, *p < 0.05, **p < 0.01, ^#^*p* < 0.001

We also investigated the relationship between MPs and cardiac FBs using the same co-culture model (Supplementary Figure 1). Givinostat had a direct action in FB cultures, increasing the expression of bone morphogenic protein 7 (bmp-7), known to be capable of inhibiting the action of TGF-β in triggering EndMT^19,20^ and decreasing p53 expression. Indeed, p53 inhibition promotes cardiac angiogenesis and reduces heart dysfunction induced by pressure damage and adaptive hypertrophy post-AMI^21^. Snail-1 and −2, which are directly regulated by tgf-β in EndMT response, were downregulated when FBs were co-cultured with MPs-M1. Finally, reduction of α-sma and increased e-cadherin expressions indicate that mesenchymal to endothelial transition (MET) was taking place, a process regulated by bmp-7. Importantly, the effect of Givinostat changes depending on whether the cells are stimulated directly or when co-cultured with MPs. In case of direct MPs-Givinostat stimulation M2 phenotype is preferred. This data confirms what is already known in the literature with reference to the wide spectrum of HDACi effects^22^. While the MP-mediated effect on CMs regards apoptosis protection (bcl2), this outcome is completely canceled if the stimulation is direct on CMs without the involvement of the inflammatory compartment. Protection against apoptosis in the CMs compartment was also confirmed in vivo, after infarction, by immunofluorescence experiments. Figure 3a shows that the positivity of CMs for TUNEL was reduced in the hearts of Givinostat treated animals at the early time points (Fig. 3a). Subsequently, after 15 and 30 days, the prevention decreased bringing the ratio similar to the control. The same effect in preventing the activation of the CM embryonic program (β-mhc, anf, gata4) in vitro, was also confirmed in vivo. We measured the cross-sectional area (CSA) of CMs both in the border and in the contralateral zones and we confirmed that the cardiac hypertrophy program was strongly reduced in the Givinostat treated group (Fig. 3b).

Interestingly, the Givinostat treatment significantly reduced the whole number of apoptotic cells in the border zone starting from day 3 up to 1 month (Fig. 3c). Histological analysis of AMI mice treated with Givinostat, also displayed an enhanced vasculature due to an increased number of α-SMA positive vessels/total area (mm^2^) at all time points (Fig. 3d) in combination with a parallel increasing in capillary density both in the border and in the contralateral zones (Fig. 3e). Another result supporting the Givinostat protective role is the increased Connexin 43 expression (CX43) in treated animals (Fig. 3f), also confirmed by western blot (Fig. 3h).

**Fig. 4:**
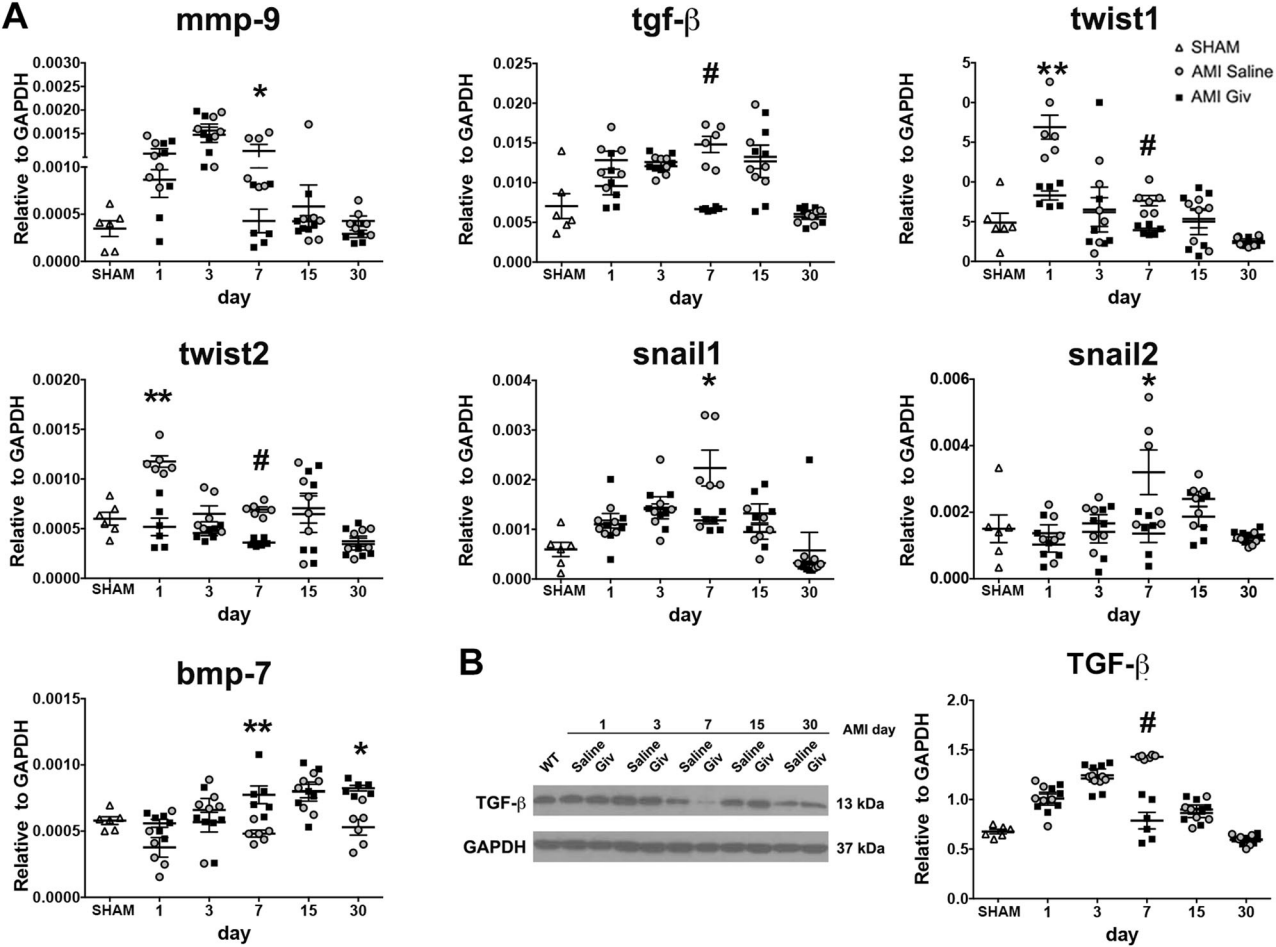
Pro-fibrotic and EndMT gene expression. **a** Quantitative RT-PCR analysis for mmp-9, tgf-β and other genes related to EndMT (twist1, twist2, snail1, snail2, bmp-7) in the whole cardiac tissue after treatment with Givinostat compared to saline injected group. **b** Western blot analysis for TGF-β. The graph indicates quantification of protein bands normalized to GAPDH in AMI Givinostat vs. AMI Saline mice at different time points. *N* = 6 mice for time point. Error bars represent ± SEM. Student’s *t* test, *p < 0.05, **p < 0.01, ^#^*p* < 0.001

Surprisingly, we noted that many of the vessels present in the hearts of the Givinostat treated animals were preserved from apoptosis after AMI, that, instead, was present in the surrounding tissue (Fig. 3g). Moreover, we observed a reduction of EndMT-related markers including a decreased gene expression of tgf-β, twist1 and 2 and snail1 and 2 and increase of bmp-7, known to counteract the fibrogenic activity of tgf-β (Fig. 4a)^20^. Coherently, TGF- β protein expression, a key regulator of cardiac fibrosis^23^, were significantly reduced compared to controls at d7 by Givinostat (Fig. 4b). A similar decrease was observed in mmp9, a pro-fibrotic marker equally involved in the pathological cardiac remodeling (Fig. 4a). Remarkably, the solely injection of Givinostat in the absence of AMI did not alter the gene expression levels of pro-fibrotic markers (supplementary Figure 2).

**Fig. 5:**
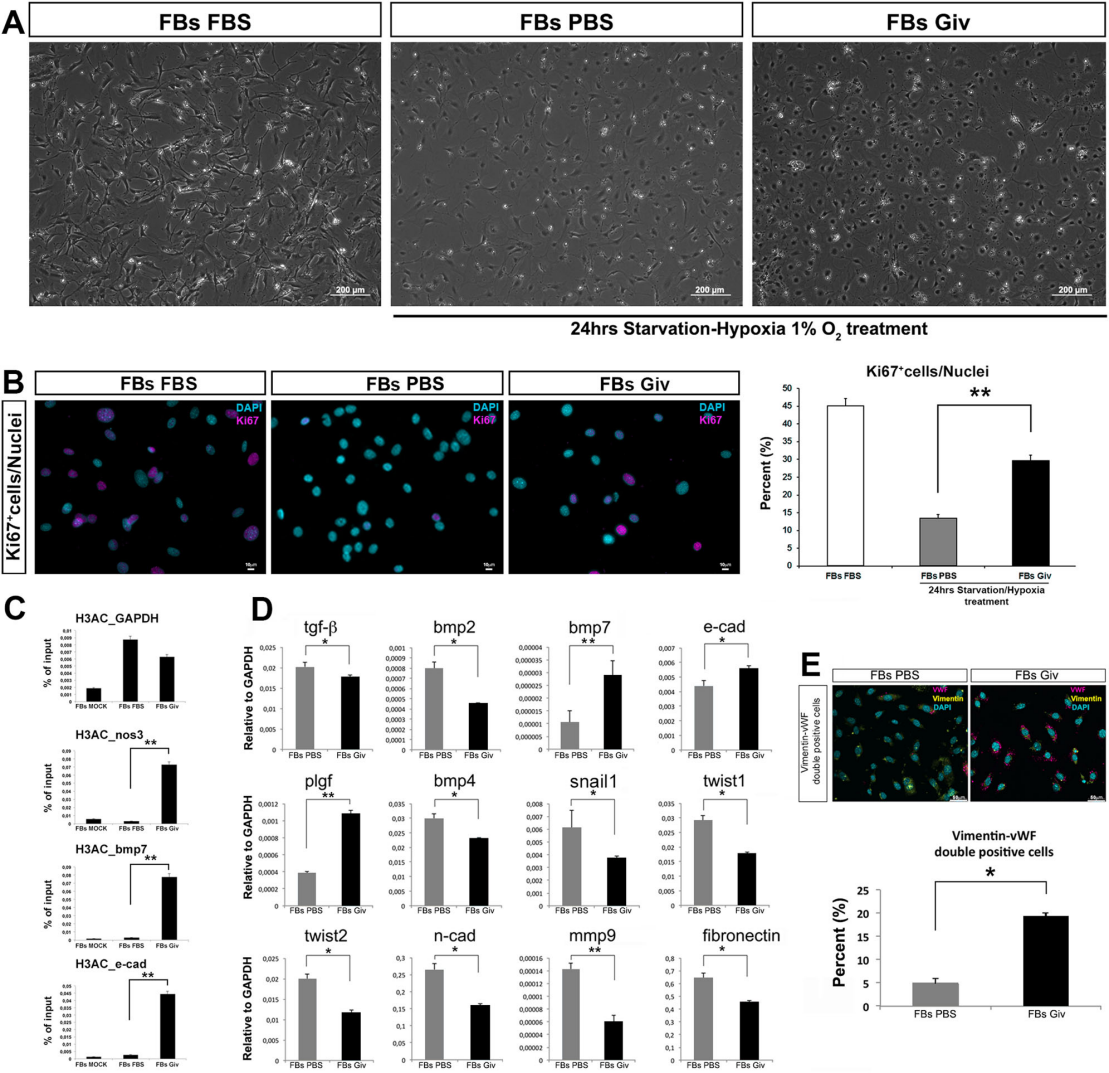
Givinostat effect on cardiac fibroblasts (FBs). **a** FBs morphology cultured in FBS supplemented media (left panel) and after starvation and hypoxia without and with Givinostat treatment (middle and right panels). Scale bar represent 200 μm. **b** Immunofluorescence for Ki67 (magenta) on FBs FBS (left panel) and after starvation and hypoxia (middle and right panels). Scale bar represent 10 μm. Graph is related to percentage of Ki67 positivity normalized to total number of nuclei (right panel). **c** ChIP assay performed in FBs treated with Givinostat or with PBS using an antibody against acetylated H3. Mock IP is included as a negative control. Graphs represent a percentage of input precipitated chromatin for nos3, bmp7, and e-cad promoters. **d** Relative gene expression related to EndMT mechanism in regards to FBs. **e** Immunofluorescence identifying double positive cells labeled for von Willenbrant Factor (vWF, magenta) and Vimentin (yellow). Scale bar represent 50 μm. The chart defines the percentage of double positive cells normalized to total cell number in FBs treated with Givinostat and the relative control. Error bars represent ± SEM. Student’s *t* test, *p < 0.05, **p < 0.01, ^#^*p* < 0.001

We than moved to an in vitro human model; human FBs were starved for 6 h and subsequently, treated with 50 nM Givinostat and exposed to 18 h of hypoxia at 1% oxygen, together with the PBS control group (Fig. 5a). The day after, cells were fixed in 4% PFA and stained for Ki67 to evaluate the proliferative capacity. The Givinostat group showed a proliferative index higher than the control, following stressful conditions (Fig. 5b). We performed a Chromatin Immuno-precipitation (ChIP) assay to detect protein-DNA interactions, among acetylation of histone 3 (H3Ac) with its target genes. The identification of the target genes and the mechanisms, by which transcription factors control gene expression, are necessary to direct the way of investigation. Specific DNA sequences were also examined by PCR. Surprisingly, we found that bmp-7, nos3, and e-cadherin^24^ promoters were associated with H3Ac confirming the active status of endothelial promoters following the treatment with Givinostat (Fig. 5c). qRT-PCR confirmed the upregulation of others endothelial genes (plgf, e-cad); in contrast, typical FB genes (n-cad, twist1 and 2, snail1, fibronectin, mmp9) were downregulated (Fig. 5d). Tgf-β, bmp-2 and -4 were also downregulated in the first 24 h while bmp-7 was more expressed making us speculate that the TGF-β-mediated EndMT mechanism was inhibited (Fig. 5d). Finally, we counted the double co-localization for Vimentin (FBs) and vWF (ECs) as transdifferentiation index.

Following hypoxic condition, FBs should activate EndMT mechanism; unexpectedly they triggered the reverse pathway known as MET after Givinostat treatment, co-expressing Vimentin and vWF in ~20% of FBs, compared to the 5% of the control (Fig. 5e).

**Fig. 6:**
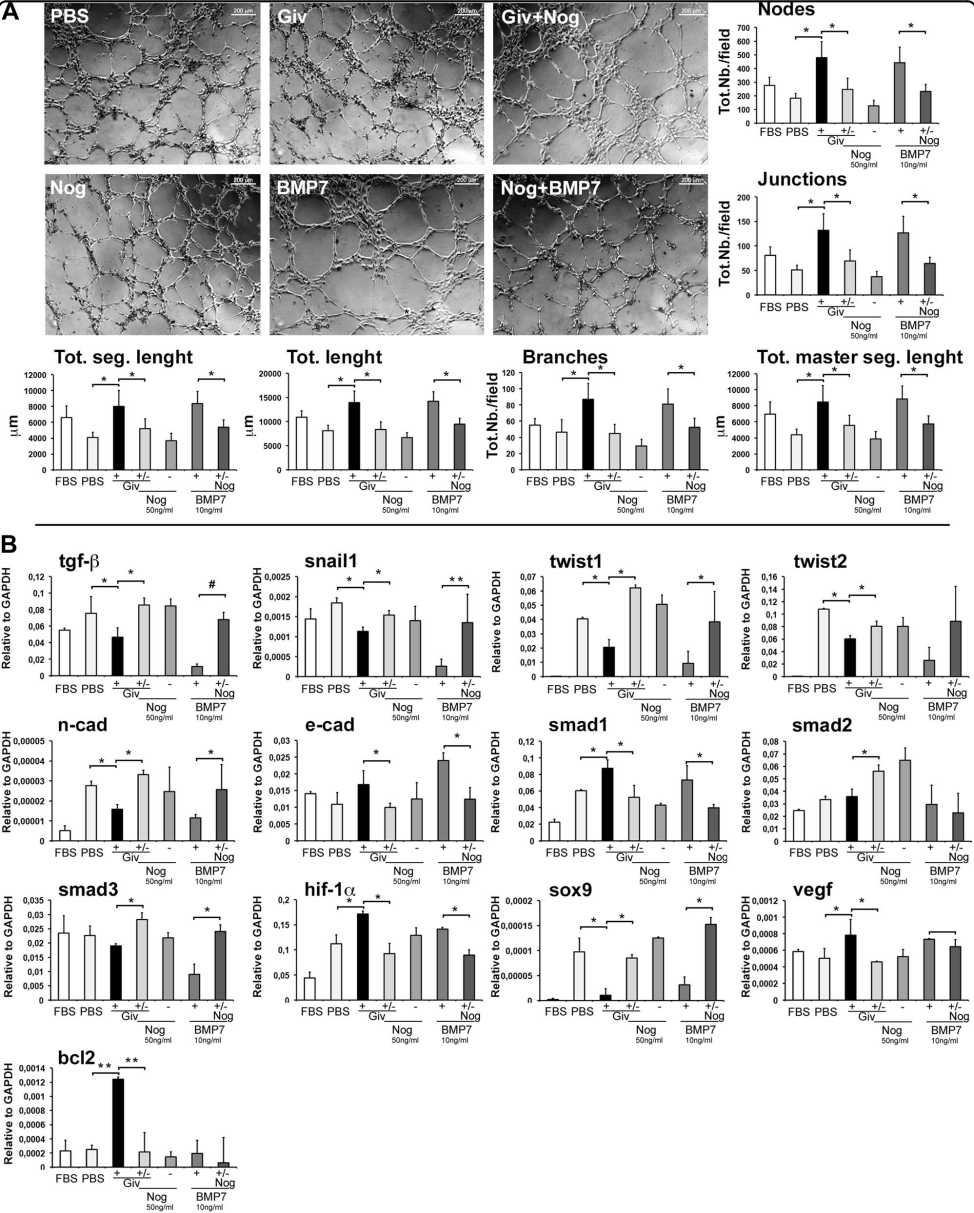
Givinostat effect on HUVECs and perturbation studies. **a** Representative images of capillary-like structures formed by HUVECs pre-treated with Noggin or BMP7 and exposed to Givinostat/FBs-conditioned media or PBS after hypoxia. Diagrams quantifying nodes, junctions, total length segments, branches, total length and total segment length formed by HUVECs using ImageJ angiogenesis software. Scale bars represent 200 μm. **b** Relative gene expression regarding EndMT mechanism (tgf-β, snail1, twist1/2, n-cad, e-cad, smad1/2/3, sox9) angiogenesis (hif-1α and vegf) and apoptosis (bcl2) in the different culture media. Error bars represent ± SEM. Student’s *t* test, *p < 0.05, **p < 0.01, ^#^*p* < 0.001

We then investigated the effect of Givinostat on human umbilical vein endothelial cells (HUVECs), which were serum-starved for 6 h and then exposed to hypoxia for 18 h. Givinostat was added to the cell cultures for the same time interval, while the control group was treated with PBS. Direct exposure to Givinostat does not alter the expression of tgf-β or bmp-7 in HUVECs (Supplementary Figure 3). Very interesting, however, it is the apoptosis protection offered by Givinostat treatment to ECs, revealed by increased bcl2 and reduced casp3 expressions (Supplementary Figure 3). Further, angiogenesis assay was performed with HUVECs exposed to supernatant harvested from starved and hypoxic FBs treated with Givinostat or PBS. Givinostat treated FBs supernatant increased HUVECs angiogenesis compared to the HUVECs directly exposed to Givinostat (Supplementary Figure 4). We performed also perturbation studies pre-treating HUVECs with Noggin (50 ng/ml) or BMP7 (10 ng/ml) and exposing them to supernatant of starved and hypoxic FBs treated with Givinostat or PBS. Noggin is a specific BMP-7 inhibitor that opposes its action in several molecular pathways included EndMT. Supernatant of FBs, starved for 6 h and exposed to 18 h of 1% oxygen hypoxia, were collected after 24 h. HUVECs culture media, after starvation, were replaced with those supernatant derived from FBs exposed to different experimental conditions. HUVECs were then exploited for angiogenesis detection test using a matrigel substrate. After 6 h we acquired the images of the cultures and analyzed by IMAGEJ integrated with Angiogenesis Software. Surprisingly, the number of knots, joints, and branches, moreover the whole total length, that of the segments and that of the master segments, increased significantly in the group exposed to supernatant of FBs treated with Givinostat and in parallel with HUVECs given BMP-7 (Fig. 6a).

qRT-PCR experiments have shown that the expression of the genes related to the EndMT mechanism are significantly reduced (Fig. 6b). In addition, the supernatant of the FBs treated with Givinostat favored the expression of genes involved in angiogenesis (e-cad, smad1, vegf) (Fig. 6b), augmenting vessel formation by an auto-looping mechanism. Very surprising was the over-expression of genes such as bcl2 (Fig. 6b), which protects ECs against apoptosis for longer-lasting angiogenic performance; in fact, tgf-β induces apoptotic cell death in HUVEC cultures down-regulating the expression of bcl-2^25^. Additionally, the over-expression of hif-1α (Fig. 6b) stimulates angiogenesis and increases blood flow^26^. Finally, sox9, a transcription factor that directly regulates the deposition of collagen type 2^27^ and the inhibition of proliferation, invasion, and EndMT in onco/plastic cells, is strongly downregulated in the treated group^28^. The results obtained from this study reveal that the beneficial effect of Givinostat is linked to the modulation of EndMT mechanism by promoting MET. On the other hand, its action is exercised in a multifactorial context, where every single cell population plays its role in contributing to the recovery of the damaged tissue.

## Discussion

Our study strengthens the rationale regarding the role of HDACi in inducing sufficient epigenetic modifications to positively remodel the heart after an ischemic event. Although first generation of HDACi (TSA, SAHA) have been already demonstrated to possess this property reducing cardiac hypertrophy and fibrosis, the molecular mechanisms have not yet been elucidated. Givinostat, similarly to other HDACi, has anti-neoplastic activity on multiple myeloma and anti-inflammatory action on several autoimmune disorders^29^. In particular, Givinostat retains anti-inflammatory effect at low concentration as opposed to TSA^30^ and its efficacy was found to be 25–50 fold more powerful than SAHA in vitro and in vivo^31^. However, the anti-fibrotic role of Givinostat in an in vivo model of heart failure has not yet been studied.

Our data highlight the role of Givinostat in preserving cardiac function following an acute cardiovascular insult. This phenomenon runs parallel to a decreased cardiac fibrosis that, together with cardiac tissue remodeling, observed earlier at day 3, exhibits premature features. This outcome is noteworthy, considering that both timing resolution and the arrest of further evolution of cardiac fibrosis after AMI represent the main key issues to positively address the healing response within the tissue^32^. To the best of our knowledge, only one study has reported the attenuation of fibrosis in presence of Givinostat, however in a model of corneal fibrosis^33^. Our results also show a reduced apoptosis in the whole cardiac muscle and an enhanced expression of Cx43, a known marker of functioning contractility^34^. The data suggests that Givinostat might act by decreasing the rate of cells loss, described to be exacerbated during infarct and negatively conditioning the compensatory re-adaptation of the cardiac muscle upon ischemic stress^35^ and in line with the ability of class I/II HDACi to control cardiac hypertrophy and foetal gene expression in CMs^36^. Interestingly, clinically approved pan HDCAi have been demonstrated able to induce cardiac-like reprogramming of stromal cells and cell death in cancer cells^37^. However, different pathophysiological disorders are capable to address diverse epigenetic assets. Thus, it is not surprising if contrasting effects are achieved by employing different HDACi.

Notably, HDACi can also target non-histone proteins therefore broadening their range of action. Besides, an enhanced vasculature^38^ was present, suggesting an additional ability of Givinostat in the adaptation of the vascular design upon cardiac hypoxia.

Our results also establish that the cardiac tissue responds to Givinostat treatment by altering its own epigenetic state. Accordingly, increased levels of acetylated Histone 3, a main relevant epigenetic modification associated with cardiovascular commitment, have been found. This correlates with the efficacy of Givinostat and is ascribable to modifications of the epigenetic state of the heart. Based on this premise, we also believe that Givinostat may exert a pleiotropic influence, resulting in an overall inhibition of the degeneration of the cardiac tissue truly due to its intrinsic deacetylation-based mechanism. Thus, the master regulators of the “maladaptive” stress response to AMI would be simply switched off or indirectly downregulated. Consequently, we investigated the gene profile and we found important cardiac tissue transcriptional and protein changes after treatment, resulting in a less fibrotic prone profile. This significant decreased in tgf-β expression was observed at day 7 in vivo, in line with the maximal tgf-β induced peak normally observed during AMI^37^. Although the trend for tgf-β levels overlap with controls on long-term, it has been recently demonstrated that cardiac protective anti-fibrotic effects due to tgf-β can be only achieved in the earliest phase of infarction^35^. Moreover, the decrease in tgf-β mRNA levels is coherent with the modulation of markers associated to a potential reduction of the EndMT phenotype^39^. Notably, the efficacy of Givinostat cannot be uncoupled from the injury. In fact, in absence of AMI transcriptional changes in the tgf-β/bmp-7 axis are not observed.

The EndMT triggered by ischemic events is one of the mechanisms that increase collagen secretion by inducing ECs to trans-differentiate into mesenchymal cells; recently it has also been shown a fundamental role of FBs in the same response, which consists in the trans-differentiation to myofibroblasts that can lead to an additional structure to the myocardium^40^. A second phase begins with an increase in fibrosis, which, once in excess, causes a stiffening of the heart wall such that systolic contraction becomes insufficient for correct blood ejection. The molecular mechanisms underlying these responses are driven by the expression of tgf-β/bmp-7 axes. Indeed, increased expression of tgf-β is capable of triggering the conversion of ECs into mesenchymal phenotype, while bmp-7 and p53 trigger the reverse process.

This study brought to light the relationship between the adjustment of this mechanism and the severity of the onset of heart failure. We have shown how Givinostat has the ability to improve heart performance by modulating the expression of tgf-β at the cardiac FB level, through a protective action against apoptosis in muscle cells and by increasing angiogenesis. The improvement of post-operative conditions after HDACi treatment is attributable to a wide-range effect of Givinostat on the different cell populations. The secretion of angiogenic key factors, by FBs, after Givinostat administration ensures an increase in vascular formation that is essential for the regenerative phase in damaged organs. We cannot ignore that the inhibition of HDACs modulates the outcomes of whole cardiac system whose sum of effects produces a benefit. The broad action of Givinostat also involves populations such as MPs and, in general, inflammatory infiltrate, which modulates behavior and exert influence on cardiac populations. We believe that the wide-ranging effect of Givinostat is its best quality. The increased angiogenesis rather than the formation of collagen in the second phase of the compensatory response guarantee an ameliorative effect that could ensure an increased life expectancy.

In conclusion, our findings underline that cardioprotection can be achieved by employing Givinostat, therefore revealing a novel clinical applicability beyond DMD.

## Materials and methods

### Animal model and surgery

The study was conducted using female C57BL/6 wild type mice (10 weeks old). Acute Myocardial infarct (AMI) was performed through permanent ligation of the left descending coronary artery as already described^41^. Animals were anesthetized with intramuscular injection of ketamine (10 mg/ml) and xylazine (1 mg/ml) and divided into two groups: (1) AMI and daily saline-only injection and (2) AMI and daily intraperitoneal injections^42,43^ of Givinostat (10 mg/Kg/d dissolved in saline solution). Both saline and Givinostat have been administered after chest closure. Mice were handled and killed (cervical dislocation) in compliance with the European Convention on Animal Care. The experimental protocol was approved and numbered as Prot. N56/2014 Ministry of Health. They have also received human cares in accordance with the guidelines from Directive 2010/63/EU of the European Parliament on the protection of animals used for scientific purposes.

### Echocardiographic measurements

Mice subjected to AMI were anesthetized by 2% isoflurane inhalation and imaged at day 0, 1, 3, 7, 15, and 30 with VisualSonics Vevo 3100® echocardiography. An M-mode scan of the left ventricle was assessed in the parasternal long axis view for measurements of intraventricular septal thickness, left ventricular posterior wall thickness, left ventricular dimension, and diastolic posterior wall velocity.

### Immunofluorescence

Hearts were perfused through abdominal aortic cannulation and fixed in Neutral-buffered Formalin 10%, dehydrated with ascending alcohols and finally included in paraffin to perform immunofluorescence on sections. Paraffin-embedded sections of 2-µm were prepared for histological analysis. The sections were dewaxed and rehydrated with descending alcohols and subjected to antigen retrieval procedure using citrate buffer pH 6.0 before staining. Slides were washed with PBS and then incubated for 30 min at room temperature with a saturating solution consisting of 10% normal Donkey serum dissolved in PBS. After blocking the sections were incubated overnight at 4 °C with the following primary antibodies used at 1:100 dilution: mouse anti- α sarcomeric actin (α-Sarc, Genetex GTX29465), anti-smooth muscle actin (α-SMA, SIGMA A2547), Connexin 43 (Cx43, Abcam, Ab3512). For Cx43 immunofluorescence images see “Method for the measurement of Connexin expression” and Supplementary Figure 5. Apoptosis was assessed by TUNEL assay (ApoAlert™ DNA Fragmentation Assay Kit, Clontech, Cat. N.630107) according to the manufacturer’s protocol. After washing with PBS, slides were incubated for 1 h with fluorescent-conjugated secondary antibodies (1:500 dilution). Negative controls were tested by incubation of only the secondary antibody without primary antibody incubation. All secondary antibodies were negative for non-specific staining. After three washes, sections were mounted with Vectashield mounting medium (VECTOR, H-1200) with DAPI.

Images were acquired by Confocal microscope TCS SP5 (Leica Microsystem). Analysis was performed in sequential scanning mode to rule out cross bleeding between channels.

Cells were fixed in 4% PFA and processed for immunofluorescence assay. Briefly, after fixation with 4% PFA for 10 min, cells were incubated overnight at 4 °C with the following primary antibodies used at 1:100 dilution: anti-Ki67 (Ki67, Abcam, 15580) anti-Vimentin (Vimentin, Abcam, 45939), anti-Von Willebrand Factor (vWF, Abcam 11713). Cells were then incubated with the appropriate secondary fluorophore-conjugated antibody. Nuclei were stained with DAPI. Images were acquired by Zeiss Microscope associated with a Nikon camera (Axio Observer A1, Zeiss, Germany).

### Method for the measurement of connexin expression

Immunofluorescence for Cx43 was converted into black and white using an image processing program ImageJ, according to the following sequence: Process>Binary>Convert to Binary. Connexin 43 quantification, area of particles, was obtained using the command Analyze>Analyze Particles (Supplementary Figure 5).

### Scar size assessment and collagen assay

Paraffin-embedded sections (2-µm) were dewaxed and rehydrated with descending alcohols and stained with Masson’s Trichrome KIT (Sigma-Aldrich, No.HT15, St. Louis, MO, USA) according to the manufacturer’s protocol. Scar size (percentage) was expressed as ratio of fibrotic area (stained in blue) on total area, using an image processing program ImageJ.

### Real time PCR

The evaluation of genes expression was performed by quantitative Real-time PCR on a 7900HT Fast Real-time PCR System equipped with SDS software (Applied Biosystems). The expression data were normalized using the Ct values of GAPDH as the housekeeping gene. Tissue samples were homogenized with Tissue Ruptor in 1 ml of TRIZOL reagent (Invitrogen, Life Technologies, 15596026). Samples homogenized were incubate for 5 min at 15 °C to 30 °C to permit a complete dissociation of nucleoprotein complexes and 0.2 ml of chloroform were added per 1 ml of TRIZOL. Tubes were vigorously shacked for 15 s and incubated at 15–30 °C to 3 min and centrifuged at 12,000 × *g* for 15 min at 4 °C. Following centrifugation, the mixture separates into three phases and RNA remains exclusively in the upper aqueous phase, the volume was about 60% of the TRIZOL volume used. The aqueous phase was transferred to a fresh tube and RNA was precipitated with 0.5 ml of isopropyl alcohol. Samples were incubated at 15–30 °C to 10 min and centrifuged 12,000 × *g* for 10 min at 4 °C. After centrifuge, supernatant was removed and RNA was visible as a pellet bottom of tube. Successively pellet was washed with 1 ml of 75% ethanol and centrifuged 7,500 × *g* for 5 min at 4 °C. At the end of procedure RNA pellet was air-dried and dissolved in 30 µl of RNasi free water. RNA quantity was determined by measuring absorbance at 260 nm using a NanoDrop UV-VIS spectrophotometer. TaqMan Fast Universal PCR Master Mix (Superscript VILO Invitrogen 11754–050) was employed to reverse transcribe RNA into single-stranded cDNA. The evaluation of genes expression was performed by quantitative real-time PCR with SYBER Green PCR Master Mix 4309155 (Applied Biosystems) on a 7900HT Fast Real-time PCR System equipped with SDS software (Applied Biosystems). Quantitative PCR parameters for cycling were set up as follows: 50 °C incubation for 2 min, 95 °C for 10 min, 40 cycles of PCR at 95 °C for 15 s, and 60 °C for 1 min (see Table 1 for primer sequences). All reactions were performed in a 15 µl reaction volume in triplicate. The expression data were normalized using the Ct values of GAPDH as the housekeeping gene.

### Western blotting

Protein samples were prepared from left ventricle and immunocomplexes were detected by chemiluminescent reaction followed by densitometry analyses with the software ImageJ. GAPDH expression levels were used to normalize the results. Tissue samples were homogenized in 500 µl of lysis buffer (50 mM TRIS HCl pH 7.5, 0.6 M sucrose, 50% glycerol, 1% TRITON, 50Mm NaCl, 10 mM NaF, 2 mM NaOV4, 1 mM PMSF, 5 mM β-glycerolphosphate, 1000× protease inhibitors) using the Tissue Ruptor disposable (4 times for 5–10 s in ice). Then, lysates were sonicated 5 s for 4 times, incubated for 20–30 min at 4 °C and centrifuged 12,000 × *g* for 15 min at 4 °C. Protein concentration was determined measuring absorbance at 598 nm using a spectrophotometer. For western blot analysis proteins were resolved by SDS-polyacrylamide gel electrophoresis and transferred to nitrocellulose membranes (0.45 µm pore size, BIO-RAD). Blots were blocked for 45 min in 5% non-fat dry milk (Sigma-Aldrich). The membranes were then incubated overnight at 4 °C with the following primary antibodies: Histone 3 (1:3000, Abcam, Ab10799) or Acetyl Histone 3 (1:3000, Merck Millipore, Cat. N.07–352), Cx43 (1:500, Cell Signaling, #3512), TGF-β (1:200, Abcam 66043). Binding of the primary antibody was detected with the use of peroxidase-conjugated secondary antibodies (1:5000, α-Rabbit NA934AV, 1:10,000 α-Mouse LNXa931/AE both GE Healthcare Life Sciences) for 1 h at room temperature. Immunocomplexes were detected by chemiluminescent reaction (ECL kit; Merck Millipore) followed by densitometry analyses with the software ImageJ. GAPDH expression levels were used to normalize the results.

### Angiogenesis assays

The effects of Givinostat-exposed fibroblasts secretomics on endothelial cell ability to form capillary-like structures on basement membrane matrix, was assessed in vitro, by morphogenesis assay. Human fibroblasts were starved for 6 h, then exposed to oxygen hypoxia 1% and subdivided in experimental groups for perturbation studies and treated with: 50 nM of Givinostat, 50 nM of Givinostat and 50 ng/ml of Noggin, 50 ng/ml of Noggin, 10 ng/ml of BMP7, 50 ng/ml of Noggin and 10 ng/ml of BMP7 for 18 h, while the control group was treated with PBS. After 24 h supernatants were collected.

HUVECs were grown on EBM2 complete medium and when 80% confluent and exposed to fibroblasts secretomics. Positive and negative controls received 10% FBS or serum free EBM2 medium, respectively. A 96-well plate, pre-chilled at −20 °C, was carefully filled with 75 μl of liquid matrigel (BD Biosciences, Milan, Italy) per well at 4 °C and let polymerized for 1 h at 37 °C. 1.5 × 10^3^ HUVEC cells/well were suspended in 100 μl of EBM2 medium containing 100 ng/ml VEGF and 100 ng/ml FGF2 alone, or with the conditioned medium and layered on the top of the polymerized matrigel. The effects on HUVECs tube formation were captured after 6 h incubation using a Zeiss Microscope associated with a Nikon camera (Axio Observer A1, Zeiss, Germany) and were quantified using ImageJ software and the “Angiogenesis Analyzer” tool.

### Bone marrow-derived macrophages

Bone marrow cells were isolated from femur and tibia of C57 mice. One million bone marrow cells were plated in 10 cm plates in 5 ml of BM-medium (DMEM supplemented with 20% low-endotoxin fetal bovine serum, 30% L929-cell conditioned medium, 1% l-glutamine, 1% Pen/Strep, 0.5% Na Pyruvate, 0.1% β-mercaptoethanol) and fed with 2.5 ml of fresh medium every 2 days. After 6 days cells were semi-confluent (80%) and used for co-culture experiments.

### Neonatal cardiomyocytes isolation

Hearts were isolated from 40 decapitated 1-to 3-day-old neonatal wild type mice with the atria dissected away were minced and digested with 108 U/ml collagenase type II (Worthington) and 0.9 mg/ml pancreatin (Life Technologies, Grand Island, NY) to obtain free cells. Myocytes were plated on gelatin-coated dishes overnight in DMEM/medium 199 (4:1) supplemented with 10% horse serum, 5% fetal calf serum, 2 mM l-glutamine (Gibco), 100 U/ml penicillin, and 100 mg/ml streptomycin (EuroClone) at a density of 1 × 10^5^ cells/cm^2^. The next day cells were rinsed three times and the plating medium was replaced with serum-free medium consisting only of DMEM/medium 199 (4:1), 2 µM l-glutamine (Gibco), 100 U/ml penicillin, and 100 mg/ml streptomycin (EuroClone); 10 µM cytosine-ß-d-furanoarabinoside was added to stop proliferation of non-cardiomyocytes and cultures contained >95% cardiac myocytes^44^. Cells were serum starved for 6 h before starting the experiments of co-culture with macrophages.

### Human fibroblasts

Stable lines of cryopreserved human cardiac fibroblasts are available in our laboratory.

### Mouse fibroblasts isolation

Hearts were isolated from 10 adult C57 mice and digested with 0.1 mg/ml of collagenase type2, 337 U/mg (Worthington) dissolved in DMEM (Gibco) at 37 °C in a shaking water bath for 10 min. The supernatant, containing free cells, was then collected and kept on ice. The digestion step was repeated three times. Cell suspensions from each digestion were pooled, filtered through a 70 μm strainer (Falcon), and centrifuged at 1200 r.p.m. for 5 min.

The cell pellet was then resuspend in a fibroblast medium (FB medium) containing high glucose DMEM (Gibco) supplemented with 10% fetal bovine serum (FBS, Invitrogen), 2 mM l-glutamine (Gibco), 100 µM NEAA (Gibco), 100 U/ml penicillin, and 100 mg/ml streptomycin (EuroClone). Cells obtained from individual animals were plated in a singular 100 mm plate in 10 ml of FB medium and incubated at 37 °C and 5% CO_2_.

### Co-culture experiments

Macrophages were plated alone or in co-culture with neonatal cardiomyocytes or cardiac fibroblasts (3:1) using a transwell system and exposed to 50 nM of Givinostat. After 1 h macrophages were polarized toward M1 phenotype with 10 ng/ml of lipopolysaccharide (LPS) or to M2 phenotype with 10 ng/ml of interleukin (IL-4), M0 macrophages were plated without polarization factors. Cells were collected in 1 ml of TRIZOL reagent after 24 h of treatment.

### Chromatin immunoprecipitation (ChIP)

Cardiac fibroblasts treated with Givinostat and the relative control, were fixed by adding directly to the culture medium formaldehyde 36.5% (Sigma Aldrich) to a final concentration of 1% and incubated for 10 min at room temperature. Then, Tris–HCl pH 7.6 was added to a final concentration of 125 mM to quench formaldehyde and cells were washed with cold PBS for 10 min on ice and rapidly collected, centrifuged at 400 × *g* for 5 min at 4 °C. Cells were lysated for 10 min in L1 buffer (50 mM Hepes-KOH pH7.5, 140 mM NaCl, 1 mM EDTA, 10% glycerol, 0.5% NP-40, 0.25% Triton X-100 supplemented with protease inhibitors) and centrifuged at 400 × *g* for 5 min at 4 °C. Cell pellets were resuspended gently in L2 buffer (10 mM Tris–HCl pH8.0, 200 mM NaCl, 1 mM EDTA, 0.5 mM EGTA) for 10 min on the wheel. After, nuclei were pelleted at 400 × *g* for 5 min at 4 °C and resuspended in L3 buffer (10 mM Tris–HCl pH 8.0, 100 mM NaCl, 1 mM EDTA, 0.5 mM EGTA, 0.1% sodium dodecyl sulfate, 0.5% N-lauroylsarcosine). Chromatin was sheared by sonication (maximum power 30 s ON, 1 min OFF, 5 min for 3 times). Chromatin IP was performed overnight on the wheel with 5 µg of Acetyl Histone 3 (Merck Millipore, Cat. N.07-352). Immunoprecipitated samples were washed six times (5 min each) with wash buffer (50 mM Hepes-KOH pH 7.6, 500 mM LiCl, 1 mM EDTA, 1% NP-40, 0.7% Na-Deoxycholate) followed by a washing in TE1X with 50 mM NaCl. Cross-linking was reversed at 65 °C overnight in elution buffer (10 mM Tris–HCl pH 8.0, 5 mM EDTA, 300 mM NaCl, 0.5%SDS), and DNA was extracted from beads by standard phenol/chloroform extraction, precipitated, and resuspended in 50 µl of H_2_O. About one-twentieth of the immunoprecipitated DNA was used in each PCR.

Quantitative real-time PCR reactions were performed in duplicate (precipitated DNA samples as well as serially diluted input DNA) with SYBER Green PCR Master Mix 4309155 (Applied Biosystems) on a 7900HT Fast Real-time PCR System equipped with SDS software (Applied Biosystems). Relative enrichment was calculated as ChIP/input ratio. Primer sequences in Table 2.

**Table 2:** Mouse and human primer sequences for quantitative RT-PCR analysis

Gene symbol	RefSeq Acc.number	Sense-forward primer	Antisense-reverse primer
MOUSE			
anf	NM_008725.2	TCTTCTTCGTCTTGGCCTTT	GACCTCATCTTCTACCGGCA
bcl2	NM_009741	GAGTACCTGAACCGGCATCT	TTGTTTGGGGCAGGTTTGTC
bmp2	NM_007553	GAAGTTCCTCCACGGCTTCT	AGATCTGTACCGCAGGCACT
bmp4	NM_007554	AGCCAACACTGTGAGGAGTT	GGATGCTGCTGAGGTTGAAG
bmp7	NM_007557	GTGGTATCGAGGGTGGAAGA	ACAAGGCCGTCTTCAGTACC
brachiury	NM_009309	GGTGCTGAAGGTAAATGTGTC	GGCTGTAATCTCTCCTCATTCTGG
col1a1	NM_007742.3	CCTCAGGGTATTGCTGGACA	GAAGGACCTTGTTTGCCAGG
col1a2	NM_007743.2	GGAACAAATGGGCTCACTGG	CAAGTCCTCTGGCACCTGTA
col3a1	NM_009930.2	CCCAACCCAGAGATCCCATT	GGTCACCATTTCTCCCAGGA
e-cadherin	NM_009864.2	CTGGACCGAGAGAGTTACCC	GTGCTTGGGTTGAAGACAGG
f4/80	NM_010130.4	CAACCTGCCACAACACTCTC	ATGAGCAGCTGTAGGATCCC
gapdh	NM_001289726	CACCATCTCCCAGGAGCGCAG	CCTTCTCCATGGTGGTGCAGAC
gata4	NM_001310610.1	GGAAGACACCTCAATCTCGT	CACAGGCATTGTACAGGTAG
il-1α	NM_010554.4	ATGTATGCCTACTCGTCGGG	CAACTCCTTCAGCAACACGG
il-1β	NM_008361.4	TGACGGACCCCAAAAGATGA	TCTCCACAGCCACAATGAGT
il-4	NM_021283	CGAGCTCACTCTCTGTGGTG	TGAACGAGGTCACAGGAGAA
mef2	NM_001170537.1	TCAGTTGGGAGTTTGCACTA	TGGTGGTACGGTCTCTAGGA
mmp9	NM_013599.4	AAAACCTCCAACCTCACGGA	GTGGTGTTCGAATGGTCTTT
mhc-α	NM_001164171.1	CCAACACCAACCTGTCCAAG	CTCGTCGTGCATCTTCTTGG
mhc-β	NM_080728.2	CCTGGAGAATGACAAGCAGC	GAGCTTCTTCTGCAGCTGAC
n-cadherin	NM_007664.4	TGGCTGAAAATAGACCCCGT	TTCCTGTCCCACTCATAGGC
nkx2.5	NM_008700	CAGTGGAGCTGGACAAAGCT	TAGCGACGGTTCTGGAATCA
p53	NM_011640	ACAGTCGGATATCAGCCTCG	GCTTCACTCGGGTCTTCAAA
plgf	NM_008827	GTTGGCTGTGCATTCCCAG	TACACCAGCTTCTCCATGGG
sma	NM_007392.3	CCTCTGGACGTACAACTGGT	GGTAGTCGGTGAGATCTCGG
snail1	NM_011427.2	CGACTACCTAGGTCGCTCTG	CTGCTGGAAGGTGAACTCCA
snail2	NM_011415.2	CGAACTGGACACACACACAG	AAAGGAGAGTGGAGTGGAGC
tnf-α	NM_013693.3	CGTCGTAGCAAACCACCAAG	GGCAGAGAGGAGGTTGACTT
tnni3	NM_009406.4	AGCAGGTGAAGAAGGAGGAC	GCATCGATATTCTTGCGCCA
twist1	NM_011658.2	GCCAGGTACATCGACTTCCT	CCAGACGGAGAAGGCGTAG
twist2	NM_007855.3	AAGATCATCCCCACGCTCC	ATTGTCCATCTCGTCGCTCT
tgf-β1	NM_011577	CAACCCAGGTCCTTCCTAAA	GGAGAGCCCTGGATACCAAC
casp3	NM_009810.3	GAGCAGCTTTGTGTGTGTGTGA	TTCGGCTTTCCAGTCAGACT
HUMAN			
bcl2	NM_000633.2	GCCCTGTGGATGACTGAGTA	GAAATCAAACAGAGGCCGCA
e-cadherin	NM_004360.3	ACAACAAGCCCGAATTCACC	GGTGTTCACATCATCGTCCG
fibronectin	NM_212482.2	CCCCATTCCAGGACACTTCT	AGGGTTCTTCATCAGTGCCA
hif-1α	NM_001530.3	ATTTTGGCAGCAACGACACA	GGGTGAGGGGAGCATTACAT
sma	NM_001141945.1	CTGCTGAGCGTGAGATTGTC	TCAAGGGAGGATGAGGATGC
mmp9	NM_004994.2	CGCTACCACCTCGAACTTTG	ATAGGGTACATGAGCGCCTC
n-cadherin	NM_001792.4	AGGGATCAAAGCCTGGAACA	TTGGAGCCTGAGACACGATT
smad1	NM_005900.2	GTACTTCCTCCTGTGCTGGT	TGGAAAAGTGGCGTTGAGTG
smad2	NM_005901.5	GACACCAGTTTTGCCTCCAG	CTCTGTGGCTCAATTCCTGC
smad3	NM_001145104.1	CTAGGGCTGCTCTCCAATGT	AAGACCTCCCCTCCGATGTA
snail1	NM_005985.3	AAGCCTAACTACAGCGAGCT	GAGTCCCAGATGAGCATTGG
snail2	NM_003068.4	AGCATTTCAACGCCTCCAAA	TGGTTGTGGTATGACAGGCA
sox9	NM_000346.3	GGCAAGCTCTGGAGACTTCT	CGCGGCTGGTACTTGTAATC
tgf-β1	NM_000660.5	CAGCAGGGATAACACACTGC	CATGAGAAGCAGGAAAGGCC
twist1	NM_000474.3	AGTCTTACGAGGAGCTGCAG	ATCTTGCTCAGCTTGTCCGA
twist2	NM_057179.2	AGAGCGACGAGATGGACAAT	CTAGTGGGAGGCGGACATG
vegf	NM_001171624.1	TCTACCTCCACCATGCCAAG	TGATGATTCTGCCCTCCTCC
casp3	NM_032991.2	AAAATACCAGTGGAGGCCGA	GCACAAAGCGACTGGATGAA
ChIP_sequences			
bmp7	NM_007557.3	GATGAGCCAGGTCCAAGAGT	AGCAACTAAGGGCTGTGCTA
e-cadherin	NM_009864.3	ACACGGAGGGAGAACAATGT	CCCCAAGTAGCAGCATCCTA
nos3	NM_008713.4	TAGGAGAGGAGCAAGGGTGA	TACAGTGGGAGGGCTTCGAG

### Statistical analysis

Statistical analysis was carried out using GraphPad (Software Inc., La Jolla, CA, USA). Values presented are mean ± SEM. Differences between sample means at each time point were evaluated with Student’s *t*-test. *P*-value of <0.05 was considered statistically significant. *P* values for each experiment are shown in supplementary information.

## Electronic supplementary material

Supplementary Figure 1

Supplementary Figure 2

Supplementary Figure 3

Supplementary Figure 4

Supplementary Figure 5

Supplementary Information
