# Supplementary figures and images for: Givinostat reduces adverse cardiac remodeling through regulating fibroblasts activation
Source: Cell Death Dis. 2018 Jan 25;9(2):108. doi: 10.1038/s41419-017-0174-5 (PMC5833837; doi:10.1038/s41419-017-0174-5)

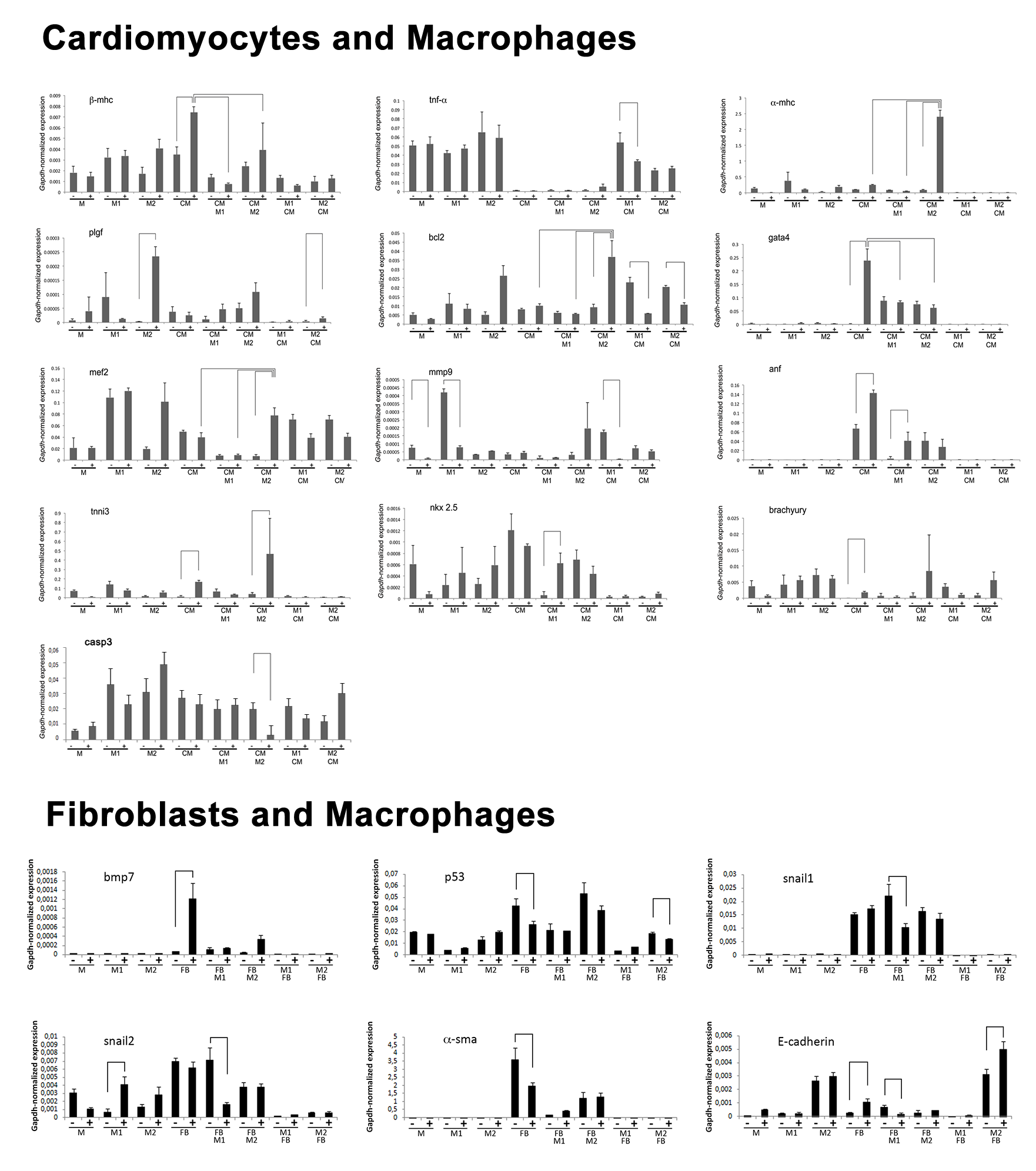

Supplement: Supplementary file 1 — Supplementary Figure 1 [file 41419_2017_174_MOESM1_ESM.tif]

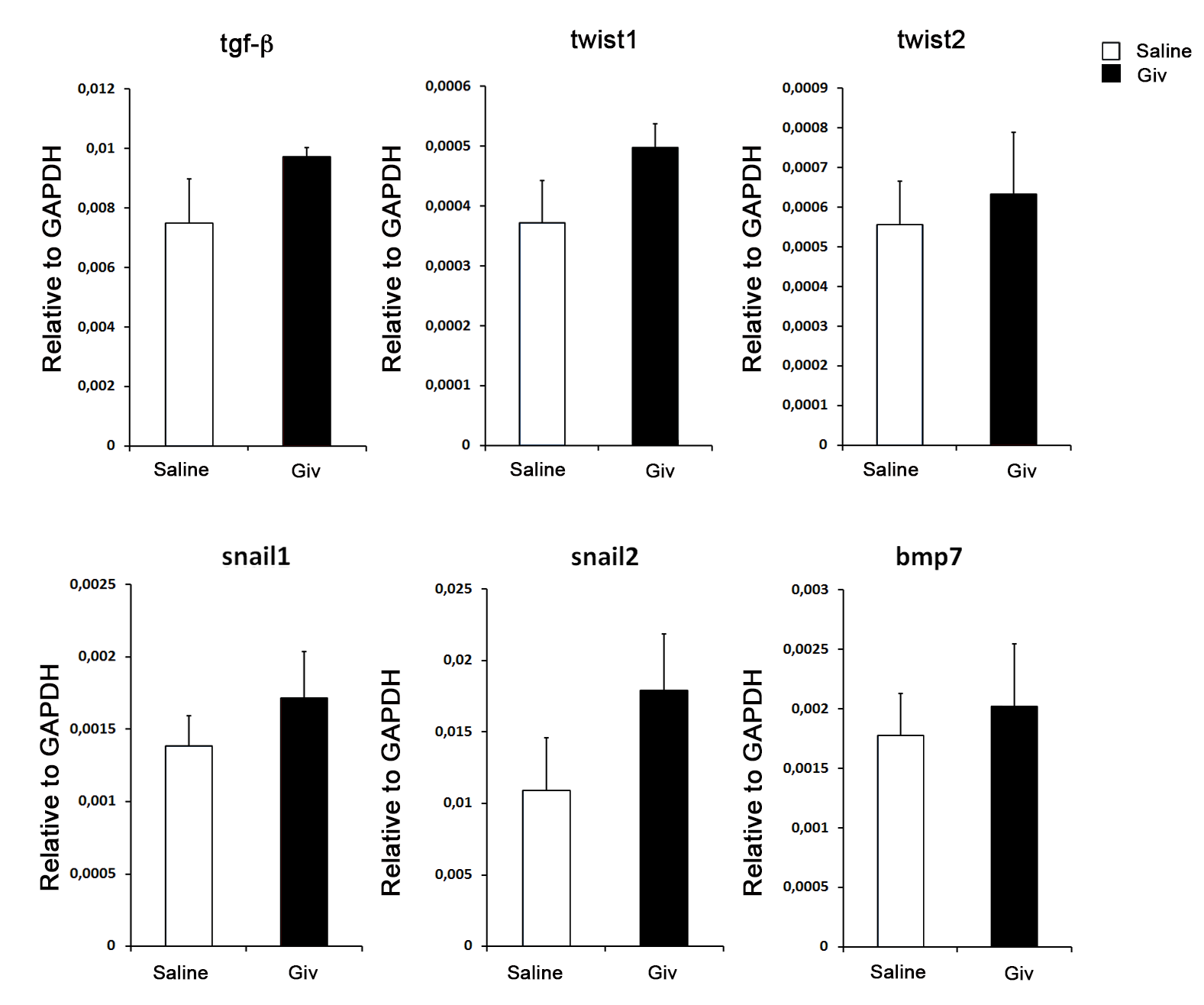

Supplement: Supplementary file 2 — Supplementary Figure 2 [file 41419_2017_174_MOESM2_ESM.tif]

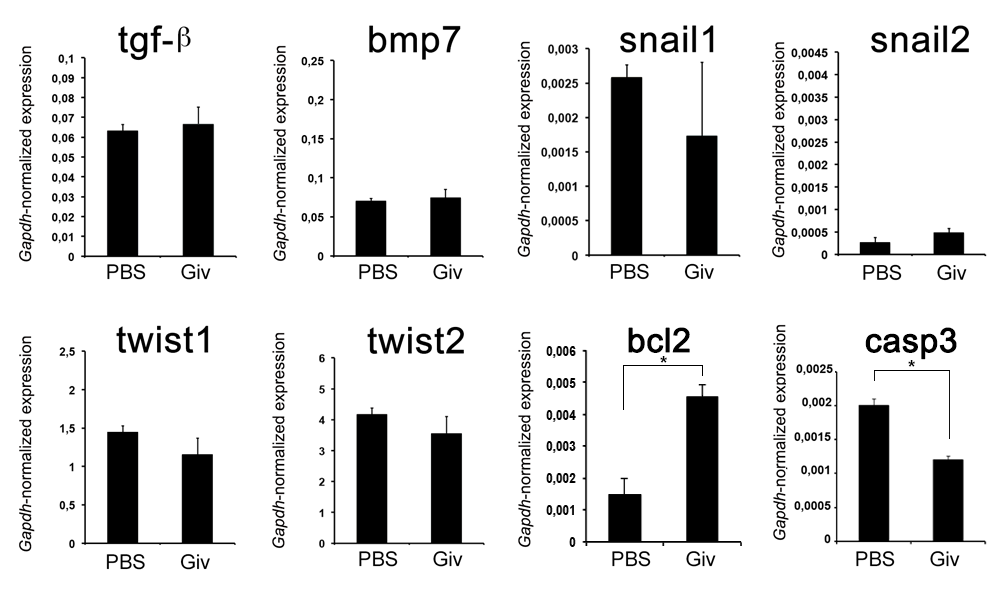

Supplement: Supplementary file 3 — Supplementary Figure 3 [file 41419_2017_174_MOESM3_ESM.tif]

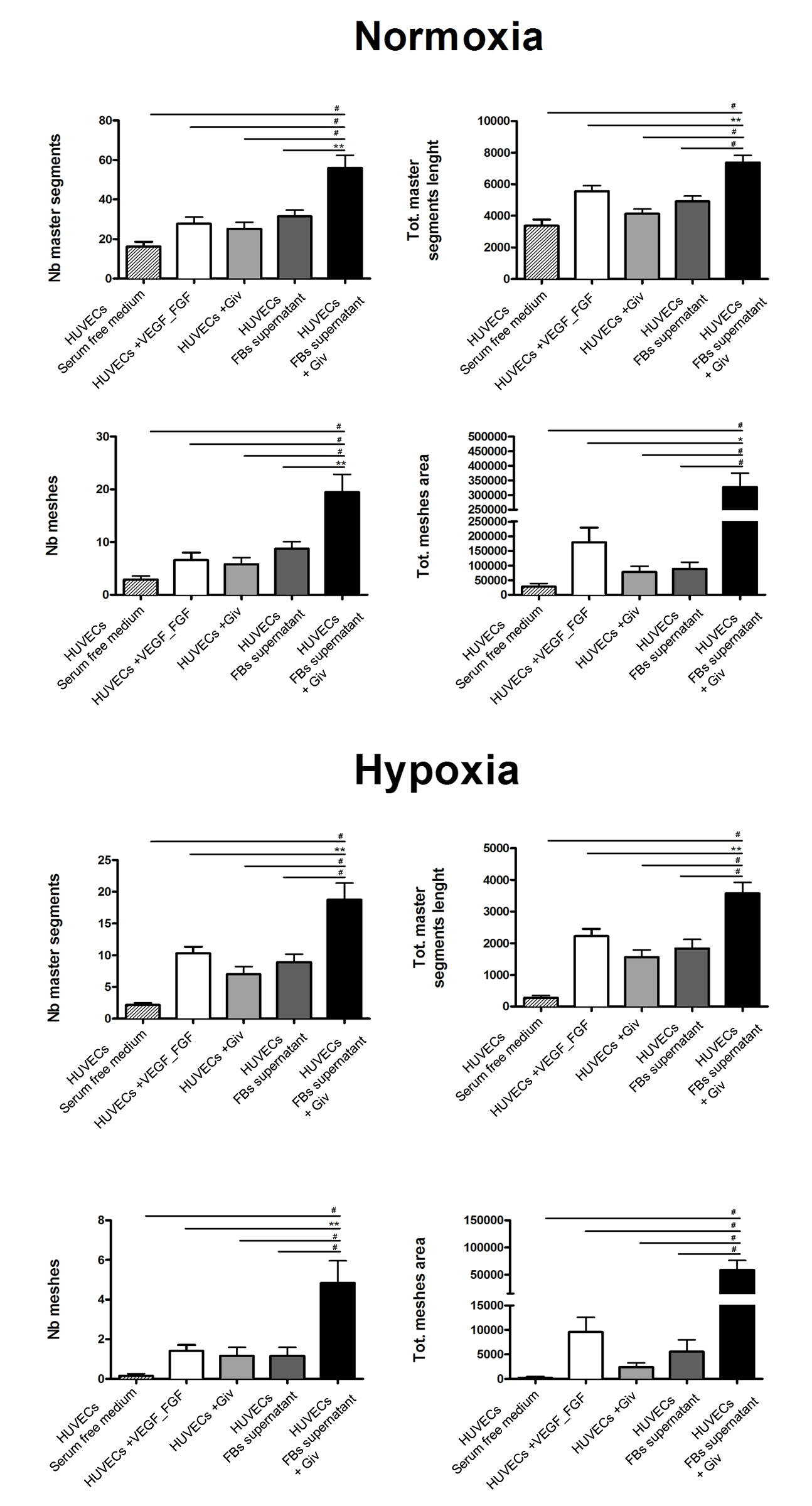

Supplement: Supplementary file 4 — Supplementary Figure 4 [file 41419_2017_174_MOESM4_ESM.tif]

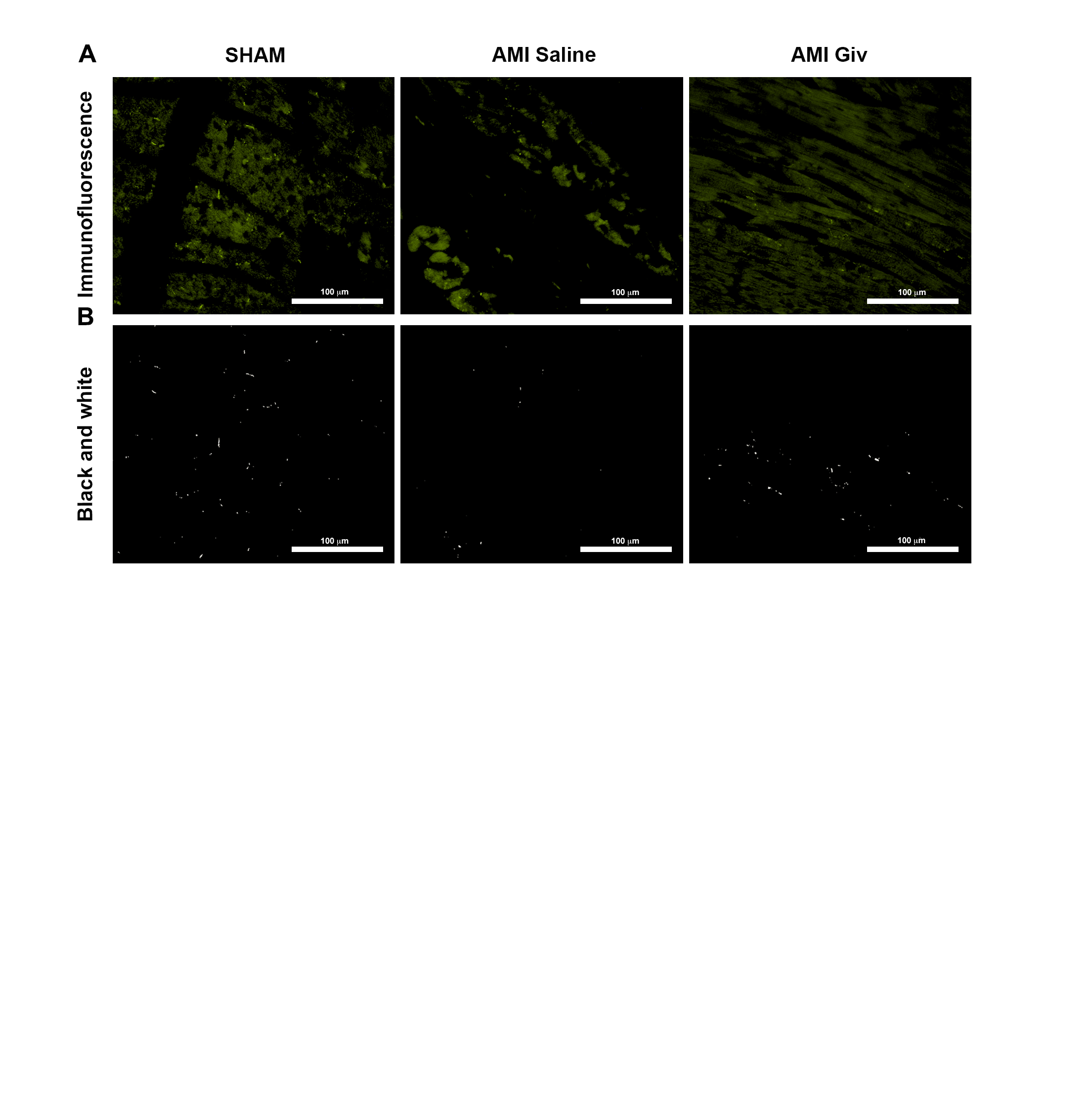

Supplement: Supplementary file 5 — Supplementary Figure 5 [file 41419_2017_174_MOESM5_ESM.tif]
