# Supplementary material for: Givinostat reduces adverse cardiac remodeling through regulating fibroblasts activation
Source: Cell Death Dis. 2018 Jan 25;9(2):108. doi: 10.1038/s41419-017-0174-5 (PMC5833837; doi:10.1038/s41419-017-0174-5)
Supplement: Supplementary file 6 — Supplementary Information [file 41419_2017_174_MOESM6_ESM.docx]

**SUPPLEMENTARY INFORMATION**

**SUPPLEMENTARY FIGURES**

**Figure S1. Co-culture experiments of neonatal cardiomyocytes or cardiac fibroblasts and macrophages induced toward M1 or M2 phenotype.** Graphs show relative gene expression related to cardiomyocytes fate, apoptosis and EndMT mechanism after exposure to vehicle or Givinostat. Error bars represent ±SEM. Student’s t test, p<0.05.

**Figure S2. Givinostat effect on mice in absence of AMI.** Charts highlight the unchanging of pro-fibrotic gene expression markers in absence of AMI and after *in vivo* administration of Givinostat in comparison to the saline injection group. Error bars represent ±SEM. Student’s t test, *p<0.05, **p<0.01, **#**p<0.001.

**Figure S3. Direct Givinostat exposure on HUVECs.** Relative gene expression related to EndMT mechanism and apoptosis on HUVECs exposed to vehicle or Givinostat. Error bars represent ±SEM. Student’s t test, *p<0.05, **p<0.01, **#**p<0.001.

**Figure S4. HUVECs angiogenesis assay.** Capillary-like structures formed by HUVECs after direct exposure of Givinostat or Givinostat/FBs-conditioned media or PBS under normoxia and hypoxia conditions. The graphs display master segments, segment length, meshes, meshes area. Error bars represent ±SEM. Student’s t test, *p<0.05, **p<0.01, **#**p<0.001.

**Figure S5.** **Cx43 quantification method in the border zone.** **(A)** Representative images of cardiac sections in the SHAM (left panel), AMI Saline (middle panel) and AMI Givinostat (right panel) 30 days post AMI. Cx43 is stained in green. **(B)** Black and white images of Cx43 staining for quantification with ImageJ as described in Materials and methods section. Scale bar represent 100μm.

**FIGURE LEGENDS p values**

**Figure 1.** **Givinostat effect on infarcted heart and cardiac fibrosis.** **(A)** FS p=0.0008 day 7, p=0.02 day 15, p=0.005 day 30; LVEDV p= 0.01 day 7, p=0.0006 day 30; LVESV p= 0.03 day 7, p= 0.0009 day 30; LVEDD p= 0.04 day 30; WT p= 0.0008 day 15. **(B)** Acetylated H3 p=6.04 x10^-8^ day 3, p=0.001 day 7, p=0.0002 day 15, p=0.04 day 30**. (C)** Fibrotic area p=0.05 day 3, p=0.003 day 7, p=0.0003 day 15, p=0.004 day 30. Col1a1 p=0.005 day 1 and 7, p=0.049 day 15; Col1a2 p=0.03 day 1, p=0.0004 day 7; Col3a1 p=0.01 day 1, p=0.004 day 7.

**Figure 2. Inflammatory process assays**. **(A)** il-1α and il-β p=0.04 day 1. **(B)** tnf-α p=0.005 and f4/80 p=0.04.**(C)** F480 p=0.04 MMP9 p=0.05.**(D)** mmp9 p=0.0002; bcl2 p=0.003, plgf p=0.0001; bmp2 p=0.004.

**Figure 3.** **Histological analysis**. **(A)** α-SARC+ / TUNEL+ cells p= 0.006 day 1, p= 0.001 day 3, p= 0.0002 day 7. **(B)** Cross-sectional area (CSA) border zone, p=0.0014 day 1, p=0.00013 day 3, p=0.002 day 7, p=0.02 day 30; contralateral zone, p=0.004 day 1, p=0.0006 day 3, p=0.001 day 7, p=0.009 day 15, p=0.003 day 30. **(C)** TUNEL-positive cells+/DAPI+ p=0.0001 day 3, p=0.0002 day 7, p=0.02 day 15, p=0.01 day 30. **(D)** α-SMA positive vessels/total area p=0.002 day 1, p=0.018 day 3, p=0.0009 day 7, p=0.02 day 15, p=1.3x10^-5^ day 30. **(E)** Capillary density border zone, p=0.03 day 1, p=0.0007 day 3, p=0.0001 day 7, p=0.0001 day 15, p=0.0019 day 30; contralateral zone, p=2.66x10-5 day 1, p= 0.001 day 3, p=1.68x10-5 day 7, p=0.001 day 15, p=0.03 day 30. **(F)** Connexin 43 p=0.004 day 1, p=0.02 day 7, p=0.01 day 15, p=0.005 day 30.

**Figure 4. Pro fibrotic and EndMT gene expression**. **(A)** mmp-9 p=0.01 day 7; tgf-β p=0.0006; twist1 p=0.004 day 1, p=0.0006 day 7; twist2 p=0.001 day 1, p=9.9x10^-5^ day 7; snail1 p=0.03 day 7; snail2 p=0.02; bmp-7 p=0.01 day 7 and 30. **(B)** TGF-β p=0.0006 day 7.

**Figure 5. Givinostat effect on cardiac fibroblasts (FBs).** **(B)** Ki67 p=0.001. (**C)** nos3 p=0.003; bmp7 p=0.002; e-cad p=0.002. **(D)** plgf p=0.005; e-cad p=0.04; n-cad p=0.045; twist1 p=0.01; twist2 p=0.03; snail1 p=0.01; fibronectin p=0.04; mmp9 p=0.005; tgf-β p=0.05; bmp2 and bmp4 p= 0.05; bmp7 p=0.002. **(E)** Vimentin and vWF double positive cells p=0.013.

**Figure 6. Givinostat effect on HUVECs and perturbation studies. (A)** Nodes p=0.021 Giv vs PBS; p=0.034 Giv+Nog vs Giv; p=0.031 Nog+ BMP7 vs BMP7; Junctions p=0.037 Giv vs PBS; p=0.047 Giv+Nog vs Giv; p=0.03 Nog+ BMP7 vs BMP7; Tot. seg. lenght p=0.009 Giv vs PBS; p=0.024 Giv+Nog vs Giv; p=0.039 Nog+ BMP7 vs BMP7; Tot. lenght p=0.04 Giv vs PBS; p=0.042 Giv+Nog vs Giv; p=0.05 Nog+ BMP7 vs BMP7; Branches p=0.04 Giv vs PBS; p=0.031 Giv+Nog vs Giv; p=0.037 Nog+ BMP7 vs BMP7; Tot. master seg. lenght p=0.02 Giv vs PBS; p=0.04 Giv+Nog vs Giv; p=0.042 Nog+ BMP7 vs BMP7. **(B)** tgf-β p=0.043 Giv vs PBS; p=0.031 Giv+Nog vs Giv; p=0.0009 Nog+BMP7 vs BMP7; snail1 p=0.02 Giv vs PBS; p=0.038 Giv+Nog vs Giv; p=0.004 Nog+ BMP7 vs BMP7; twist1 p=0.02 Giv vs PBS; p=0.02 Giv+Nog vs Giv; p=0.04 Nog+ BMP7 vs BMP7; twist2 p=0.01 Giv vs PBS; p=0.034 Giv+Nog vs Giv; n-cad p=0.02 Giv vs PBS; p=0.019 Giv+Nog vs Giv; p=0.049 Nog+BMP7 vs BMP7; n-cad p=0.02 Giv vs PBS; p=0.019 Giv+Nog vs Giv; p=0.049 Nog+BMP7 vs BMP7; e-cad p=0.03 Giv+Nog vs Giv; p=0.025 Nog+ BMP7 vs BMP7; smad1 p=0.02 Giv vs PBS; p=0.035 Giv+Nog vs Giv; p=0.035 Nog+BMP7 vs BMP7; smad2 p=0.03 Giv+Nog vs Giv; smad3 p=0.02 Giv+Nog vs Giv; p=0.04 Nog+BMP7 vs BMP7; hif-1α p=0,03 Giv vs PBS; p=0.035 Giv+Nog vs Giv; p=0.02 Nog+BMP7 vs BMP7; vegf p=0.04 Giv vs PBS; p=0.01 Giv+Nog vs Giv; p=0.05 Nog+BMP7 vs BMP7; bcl2 p=0.001 Giv vs PBS; p=0.009 Giv+Nog vs Giv.
